# Acupuncture attenuates myocardial ischemia/reperfusion injury-induced ferroptosis via the Nrf2/HO-1 pathway

**DOI:** 10.1186/s13020-025-01114-0

**Published:** 2025-05-09

**Authors:** Xiao Li, Yu-xin Sun, Adi Wirawan Tjahjono, Ying Wei, Xiang Li, Qian-hua Zheng, Wen-chuan Qi, Fan-rong Liang

**Affiliations:** 1 School of Acu-Mox and Tuina, Chengdu University of Traditional Chinese Medicine, Chengdu, 610075 People’s Republic of China; 2 School of Clinical Medicine, Chengdu University of Traditional Chinese Medicine, Chengdu, 610075 People’s Republic of China; 3 Sichuan Provincial Key Laboratory of Acupuncture and Chronobiology, Chengdu, 610075 People’s Republic of China

**Keywords:** Myocardial ischemia–reperfusion injury, Acupuncture, Ferroptosis, NFE2-related factor 2, Heme oxygenase-1

## Abstract

**Aims:**

To observe the effect of electro-acupuncture (EA) on cardiomyocytes ferroptosis induced by myocardial ischemia/reperfusion injury (MIRI) in mice and to investigate whether this effect occurs via the nuclear factor-E2-related factor 2 (Nrf2)/heme oxygenase 1 (HO-1) signalling pathway.

**Materials and methods:**

Firstly, Fe^2+^ in the hearts and serum of mice from both the sham-operated (SO) group and MIRI group was measured to ascertain whether ferroptosis had occurred in the cardiomyocytes of mice in MIRI group. In the second phase, EA was administered, with sham acupuncture (SA) group as the comparator, to investigate the protective effects of EA on ferroptosis in MIRI cardiomyocytes and cardiac function. Additionally, we studied the levels of Nrf2 and HO-1 within the myocardium. In the third phase, Nrf2 inhibitor ML385 and agonist DMF were applied to observe the impact of inhibiting Nrf2 on the therapeutic efficacy of EA.

**Results:**

Compared with SO group, MIRI group showed increased iron deposition, along with a significant decrease in Nrf2 and HO-1 levels. Compared with MIRI group, MIRI + EA group exhibited significantly improved cardiac function and reduced cardiac iron deposition, accompanied by increased Nrf2 and HO-1 levels. Furthermore, the therapeutic effect of MIRI + EA group was superior to that of MIRI + SA group. Administration of ML385 partially blocked the anti-ferroptotic and cardioprotective effects of EA, while EA treatment exhibited similar effects to dimethyl fumarate (DMF) intervention.

**Conclusion:**

EA alleviates ferroptosis-induced damage in MIRI in mice via the Nrf2/HO-1 pathway, providing modern scientific evidence for the application of acupuncture in the treatment of cardiovascular diseases.

**Supplementary Information:**

The online version contains supplementary material available at 10.1186/s13020-025-01114-0.

## Introduction

Myocardial ischemia/reperfusion (IR) is an effective treatment for acute myocardial infarction. However, myocardial ischemia/reperfusion injury (MIRI), which occurs during this process, has become a significant factor affecting therapeutic outcomes and patient prognosis [1]. Recent studies have revealed that MIRI can induce ferroptosis in cardiomyocytes, a form of cell death caused by iron-dependent lipid peroxidation, resulting in severe damage to myocardial cells [2, 3]. Studies have shown that effectively inhibiting cardiomyocyte ferroptosis induced by reperfusion can help ameliorate MIRI-related cardiomyocyte damage and the decline in cardiac function [4, 5]. Previous researches have found that acupuncture, as a traditional Chinese medical therapy with flexible intervention timing, diverse therapeutic protocols, and fewer side effects, can alleviate myocardial injury and improve cardiac function [6–8], as well as reducing cell death [9, 10] and inhibiting inflammatory responses [11]. Moreover, acupuncture can alleviate myocardial injury by reducing oxidative stress level [12] and regulating various antioxidant pathways [13]. Acupuncture is also associated with iron metabolism in many diseases [14, 15]. However, it remains unclear whether acupuncture can ameliorate MIRI by mitigating cardiomyocyte ferroptosis.

Nuclear factor-E2-related factor (Nrf2), an essential oxidative stress response regulator, protects cells from oxidative stress and inflammatory responses. Under oxidative stress stimulation, Nrf2 translocates from the cytoplasm to the nucleus, where it binds to the antioxidant response element (ARE), activating the expression of downstream antioxidant enzymes and anti-inflammatory proteins such as heme oxygenase-1 (HO-1) [16]. HO-1, a key antioxidant enzyme, catalyses the degradation of heme into biliverdin, carbon monoxide, and ferrous ions, thereby exerting antioxidant, anti-inflammatory, and anti-apoptotic effects [17]. Research has demonstrated that the Nrf2/HO-1 pathway is intricately linked to the generation of reactive oxygen species (ROS), the accumulation of lipid peroxides, and iron metabolism [18]. Consequently, it is recognized as a pivotal target for inhibiting MIRI-induced ferroptosis [18] and is also implicated in a spectrum of cardiac diseases, including MIRI [19, 20]. However, whether acupuncture inhibits MIRI-induced ferroptosis via the Nrf2/HO-1 pathway remains unclear. Therefore, this study aims to investigate the effects of acupuncture on cardiomyocyte ferroptosis induced by MIRI in mice and explore whether these effects are mediated through the Nrf2/HO-1 signalling pathway.

## Materials and methods

### Animals and ethics

Healthy specific pathogen-free (SPF) male Kunming mice, weighting 30–34 g, were provided by Chengdu Dashuo Laboratory Animal Technology Co., Ltd. (License No. SCXK 2020–030). The animals were reared in the experimental animal research center in Chengdu University of Traditional Chinese Medicine in a barrier environment (License No. SYXK 2024–0049). Environmental conditions were maintained at 20–24 °C, relative humidity of 45%−55%, and a 12 h light/dark cycle. The bedding was replaced every other day, and the mice were provided with standard laboratory feed and water ad libitum. The mice were acclimatised to the environment for 7 days prior to the commencement of experiments. This study was approved by Chengdu University of Traditional Chinese Medicine Animal Welfare and Ethics Committee (Approval No. 02022–01), and all experimental procedures conformed to the *Guide for the Care and Use of Laboratory Animals* by the National Institutes of Health (1996).

### Experimental design

In the first phase, 14 mice were randomly allocated, with 6 assigned to the sham operation (SO) group and 8 to the model group. Following modelling, 2 mice died, leaving 6 mice in the MIRI group.

In the second phase, 31 mice were randomly assigned using a random number table, with 6 allocated to the SO group and 25 to the model group. After modelling, 7 mice died. The remaining 18 successfully modelled mice were randomly divided into the MIRI group, MIRI + electro-acupuncture (EA) group, and MIRI + sham acupuncture (SA) group, with 6 mice per group.

In the third phase, 46 mice were randomly assigned using a random number table, with 6 allocated to the SO group and 40 to the model group. Following modelling, 10 mice died. The remaining 30 successfully modelled mice were randomly divided into the MIRI group, MIRI + EA group, MIRI + EA + ML385 group, MIRI + dimethyl fumarate (DMF) group, and MIRI + vehicle group, with 6 mice in each group.

**Fig. 1:**
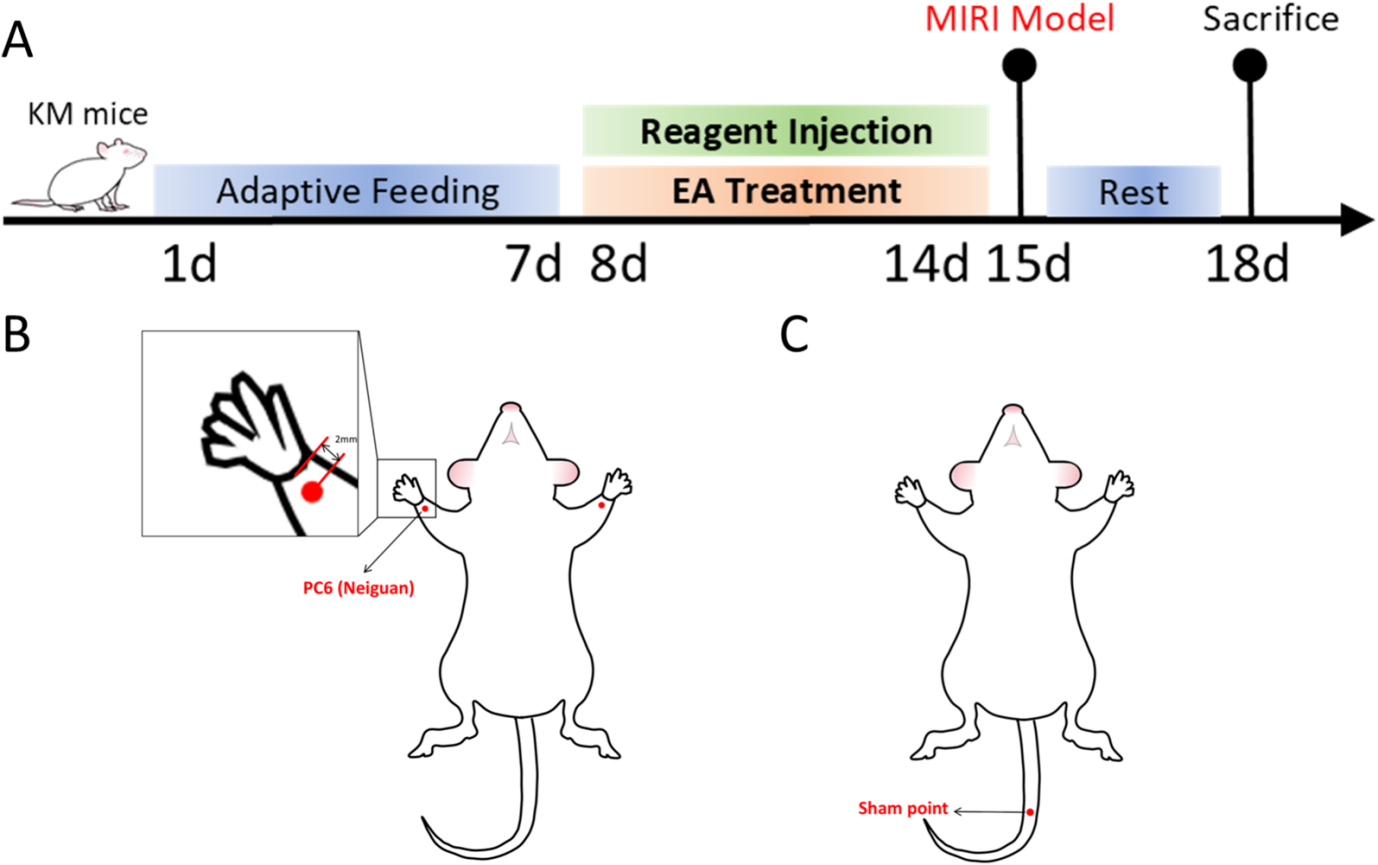
Experiments protocols and acupoint locations. **A** Experiments protocols. **B** Location of PC6 (Neiguan) acupoint. **C** Location of the sham point

The experimental workflow is shown in Fig. 1A.

### Animal models

After fasting for 12 h, the mice were anesthetized with 2% isoflurane (RWD, China). The left chest was depilated and a small incision (about 1.2 cm) was made. A 3–0 silk thread was inserted to prepare for suturing. After dissecting the muscle, the fourth intercostal space was exposed, and a hole was made using mosquito forceps to open the cardiothoracic membrane. The heart was gently"popped up", and a 6–0 silk thread was used to locate and ligate the left coronary artery (LCA) approximately 3 mm from its origin. Successful ligation was confirmed when the anterior wall of left ventricle turned white, after which the heart was returned to its original position. The thoracic cavity was promptly closed to expel air and reduce tension. Following the operation, the mice were placed on a 37 °C thermal pad with respiratory monitoring. After 20 min, the ligature silk thread was gently removed, the skin incision was sutured, and the procedure was completed [21]. The SO group underwent the same procedure without LCA ligation. 72 h after modelling, the mice were anaesthetised with pentobarbital sodium (40 mg/kg; Sigma, USA), and samples were collected for subsequent tests.

### EA pre-treatment

Seven days prior to modelling, mice in the MIRI + EA group and MIRI + SA group received daily electroacupuncture for 20 min per session. The mice were placed in the supine position and anaesthetised using 2% isoflurane for induction, followed by 1% isoflurane to maintain anaesthesia. In the MIRI + EA group, bilateral PC6 acupoints (located on the medial side of the forelimb, approximately 2 mm proximal to the wrist joint, Fig. 1B) were alternately stimulated with electroacupuncture. The needles (0.25 mm × 13 mm; Huatuo, China) were inserted 2–3 mm into the PC6 acupoint, and an auxiliary needle was placed approximately 3 mm below the point along the pericardium meridian at a depth of 1–2 mm. In the MIRI + SA group, a needle was inserted into a non-acupoint at the tail (a point located at the midline of the tail as a control, Fig. 1C). The needle was inserted 2–3 mm deep, and an auxiliary needle was placed approximately 3 mm distal to the point at a depth of 1–2 mm. The acupuncture needles and auxiliary needles were connected to the positive and negative electrodes of SDZ-3 electroacupuncture instruments (Hua Tuo, China). The device was set to a continuous wave with a frequency of 2 Hz, and the current was adjusted until mild twitching of the forelimbs or tail was observed in the mice.

### Reagent injection

In the MIRI + SA + ML385 group, mice were administered an intraperitoneal injection of the Nrf2 inhibitor ML385 (30 mg/kg; Selleck, USA) 1 h prior to acupuncture each day. ML385 was prepared in a solution consisting of 6% dimethyl sulfoxide (DMSO), 40% polyethylene glycol 300 (PEG 300, Selleck, USA), 5% Tween 80 (Selleck, USA), and 49% double-distilled water (ddH₂O) [22]. In the MIRI + DMF group, mice were administered the Nrf2 activator dimethyl fumarate (DMF; 10 mg/kg; Selleck, USA) via daily intraperitoneal injection. DMF was prepared in a solution of 10% DMSO, 40% PEG 300, 5% Tween 80, and 45% ddH₂O [23]. In the MIRI + vehicle group, mice were injected daily with an equivalent volume of the ML385 vehicle solution.

### Cardiac ultrasound

Mice were anaesthetised with 2% isoflurane, and the fur on the anterior chest area was removed. Conductive gel was applied to the chest, and the heart was located using an ultrasound imaging system (Mindray, China). The ultrasound probe was placed on the mouse’s chest to perform a layer-by-layer scan of the cardiac tissues. Using M-mode echocardiography, the left ventricular ejection fraction (EF) and fraction shorting (FS) were measured and recorded for 3 cardiac cycles per mouse. The average of the three cardiac cycles was calculated and used as the final result. The cardiac ultrasound examination conducted in this study was performed by a trained and professional ultrasound technician.

### Cardiac magnetic resonance examination

Using a GE Optima 360 W 1.5 T MR scanner, late gadolinium enhancement (LGE) and T2* sequences were performed with electrocardiogram and respiratory gating. An eight-echo sequence was acquired during a single breath-hold, with scanning performed in the short-axis plane of the left ventricle from the apex to the base. All measurements were processed and analysed 3 times using the GE Advantage Workstation 4.6, with the average value calculated. All examinations were conducted by a trained and experienced radiologist proficient in small animal magnetic resonance examinations. In cases where significant variations were observed among the three measurements, another radiologist was consulted to re-measure and re-assess the methodology.

### Measurement of myocardial enzyme and inflammatory factor

Serum was collected for the analysis of inflammatory factors including interleukin-1β (IL-1β), interleukin-6 (IL-6), and tumour necrosis factor-α (TNF-α), as well as myocardial enzyme levels, including creatine kinase (CK), creatine kinase-MB (CK-MB), and lactate dehydrogenase (LDH). Heart tissue samples were also collected to measure myocardial inflammatory factor levels (IL-1β, IL-6, and TNF-α). Enzyme-linked immunosorbent assay (ELISA) kits (Elabscience, China) were used for the detection of IL-1β, IL-6, TNF-α, CK, and CK-MB, following the manufacturer’s instructions. LDH levels were measured using colourimetric assay kits (Elabscience, China) in accordance with the manufacturer’s protocols.

### Serum and myocardial Fe^2+^ determanation

Serum and heart tissue samples were collected to measure Fe^2+^ levels. A Fe^2+^ colorimetry kit (Elabscience, China) were used in accordance with the manufacturer’s instructions.

### Malondialdehyde (MDA) detection

Serum was collected to measure MDA levels. An MDA colorimetry kit (Elabscience, China) was used in accordance with the manufacturer’s instructions.

### Glutathione peroxidase 4 (Gpx4) detection

Heart tissue samples were collected collected to measure Gpx4 levels. An Gpx4 ELISA kit (Nanjing Jiancheng Bioengineering, China) was used in accordance with the manufacturer’s instructions.

### Cardiac histopathology with Hematoxylin–eosin (HE) staining

The heart was fixed in 4% neutral paraformaldehyde solution (Solarbio, China) following extensive flushing with 1 × PBS (Gibco, USA). The tissue was cut into appropriately sized pieces, washed, dehydrated and embedded in paraffin wax. The samples were then sectioned into 3–4 µm thick slices and stained with hematoxylin and eosin (Leigen, China) in accordance with the manufacturer’s protocols. Images of the stained sections were captured using a microscope (Nikon, Japan).

### ROS detection with flow cytometry

A cell suspension was prepared using 20 mg of fresh heart tissue. DCFH-DA (Beyotime, China) was diluted to a final concentration of 10 µmol/L, and the diluted DCFH-DA was added to each sample, adjusting the cell concentration to 1–20 × 10⁶ cells/mL. The samples were incubated in a 37 °C cell incubator for 20 min, with gentle mixing by inversion every 3–5 min. The cells were then washed three times with serum-free cell culture medium, collected, and subjected to fluorescence signal detection using a flow cytometer. The fluorescence spectrum of DCF is highly similar to that of FITC; therefore, the parameters for FITC were used to detect DCF. Data analysis was performed using FlowJo V10.8.1 (BD Biosciences, USA).

### Double immunofluorescence staining

Heart tissue samples were fixed in 4% neutral paraformaldehyde solution (Solarbio, China) for at least 2 h after dissection. The tissues were then cut into appropriately sized pieces, washed, dehydrated, embedded, and sectioned into 3 µm thick slices. For staining, antigen retrieval was performed using a citric acid antigen retrieval solution (Solarbio, China). The sections were incubated with primary antibodies, including a rabbit recombinant anti-Nrf2 antibody (1:300; Proteintech, China; 80,593–1-RR) and a rabbit polyclonal anti-HO-1 antibody (1:200; Proteintech, China; 10,701–1-AP). DAPI (Solarbio, China) was used to stain the nuclei, and the sections were washed with PBS and sealed with an autofluorescence quencher (Sudan Black B; Sangon Biotech, China) and anti-fluorescence quenching sealing tablets (Southern Biotech, USA). Images were captured using a fluorescence microscope (Nikon, Japan), and three random fields of view were selected from each sample for analysis using CaseViewer 2.4 image software (3D Histech, Hungary). Quantitative analysis of Nrf2 + cells and HO-1 + cells was performed using Aipathwell software, with the average value from three fields of view used as the final result for each sample.

### Western blotting

Proteins were extracted from heart tissue samples stored at − 80 °C. The concentration of extracted proteins was measured and calculated using a BCA protein assay kit (Beyotime, China) according to the manufacturer’s instructions. Protein concentrations in each sample were adjusted to 3 µg/µL using 5 × SDS loading buffer and lysis buffer, followed by boiling in a metal bath. The proteins were separated by electrophoresis on a 12% SDS-PAGE gel (Biosharp, China) and transferred onto PVDF membranes (Servicebio, China) using standard experimental procedures. The membranes were incubated with the following primary antibodies: anti-Nrf2 antibody (1:3000; Thermo Fisher, USA; PA5-27,882), anti-HO-1 antibody (1:5000; Abcam, UK; ab189491), anti-mitochondrial ferritin (FtMt) antibody (1:1000, Thermo Fisher, USA; PA5-30,906), and anti-β-actin antibody (1:5000; Affinity, China; AF7018). Detection was performed using a hypersensitive ECL chemiluminescent substrate and visualised with a chemiluminescence imaging system (ChemiScope, China). Data analysis was conducted based on the captured images.

### Quantitative real-time polymerase chain reaction

A total of 10 mg of heart tissue was used for total RNA extraction with Trizol (Foregene, China). The RNA concentration was determined, and all RNA samples were adjusted to a concentration of 500 ng/µL before reverse transcription into cDNA (ABM, Canada). Using β-actin as an internal control, target gene expression was analysed with specific forward and reverse primers (Tsingke, China) via quantitative real-time polymerase chain reaction (qRT-PCR). Each sample was tested in triplicate wells. The Ct values were obtained using CFX Manager 3.1 software, and the relative expression of target genes was calculated using the 2^−△△Ct^ method. The primer sequences are shown in Table 1.

**Table 1:** Real-time PCR primer sequences

Gene	Primer sequences (forward)	Primer sequences (reverse)
Nrf2	GCCACCGCCAGGACTACAG	AACTTGTACCGCCTCGTCTGG
HO-1	AAGACCGCCTTCCTGCTCAAC	TCTGACGAAGTGACGCCATCTG
β-actin	GTGCTATGTTGCTCTAGACTTCG	ATGCCACAGGATTCCATACC

PCR, polymerase chain reaction

### Statistical analysis

All data are expressed as the mean ± standard deviation (SD) and were analyzed using GraphPad Prism Software version 8.0 (GraphPad, La Jolla, California, USA). The significance of differences between two groups was determined using Student’s unpaired t-test, while differences among more than two groups were evaluated using one-way ANOVA test followed by the Holm-Sidak test. Statistical significance was defined as p < 0.05, and high statistical significance as p < 0.01. p values for all comparisons are indicated in the graphs, with “ns” (non-significant) used for statistically insignificant results. Full statistical details of the experiments can be found in the figure legends.

## Results

### MIRI induces ferroptosis in cardiomyocytes

**Fig. 2:**
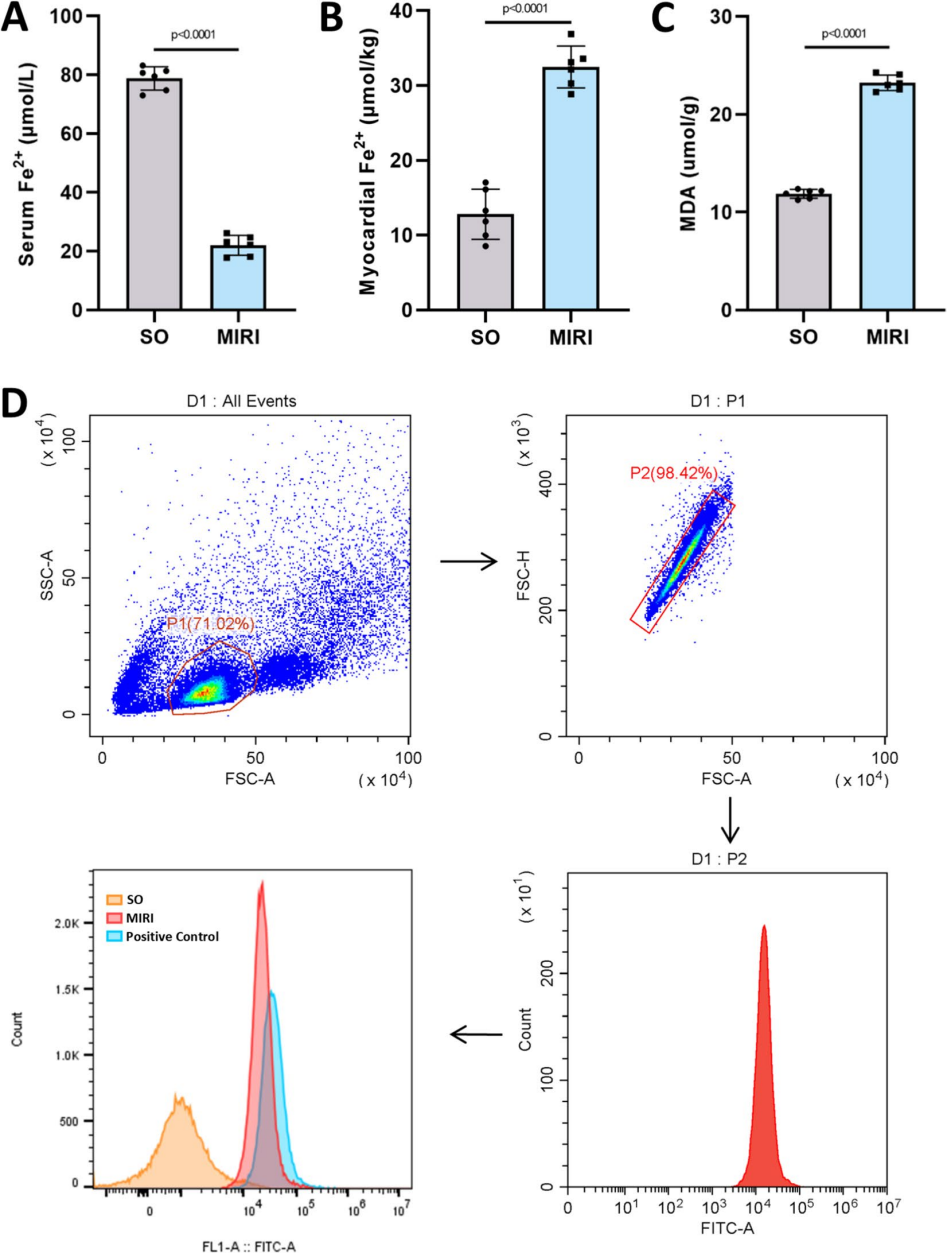
Myocardial ischemia/reperfusion injury (MIRI) induced cardiomyocyte ferroptosis. **A** Content of Fe^2+^ in serum of sham operation (SO) and MIRI mice; n = 6; **B** Content of Fe^2+^ in myocardium of SO and MIRI mice; n = 6; **C** Serum malondialdehyde (MDA) levels in SO and MIRI mice; n = 6; **D** Expression of reactive oxygen species (ROS) in the myocardium of mice in SO and MIRI mice detected by flow cytometry, and the gating strategy and analysis procedure were displayed. p values for all comparisons are indicated in the graph

Previous studies have demonstrated that MIRI induces ferroptosis in cardiomyocytes [24, 25]. To confirm that our MIRI model induces ferroptosis in cardiomyocytes, we measured ferroptosis-related markers in serum and myocardial tissue prior to the formal experiments. The results showed that, compared with the SO group, serum Fe^2^⁺ levels were significantly reduced in the MIRI group (Fig. 2A), while myocardial Fe^2^⁺ levels were significantly elevated (Fig. 2B). Additionally, following MIRI, lipid peroxidation product malondialdehyde (MDA) levels in the serum were increased (Fig. 2C), and intracellular ROS levels in cardiomyocytes were significantly elevated (Fig. 2D).

### Acupuncture alleviates cardiomyocyte ferroptosis and reduces myocardial iron deposition

**Fig. 3:**
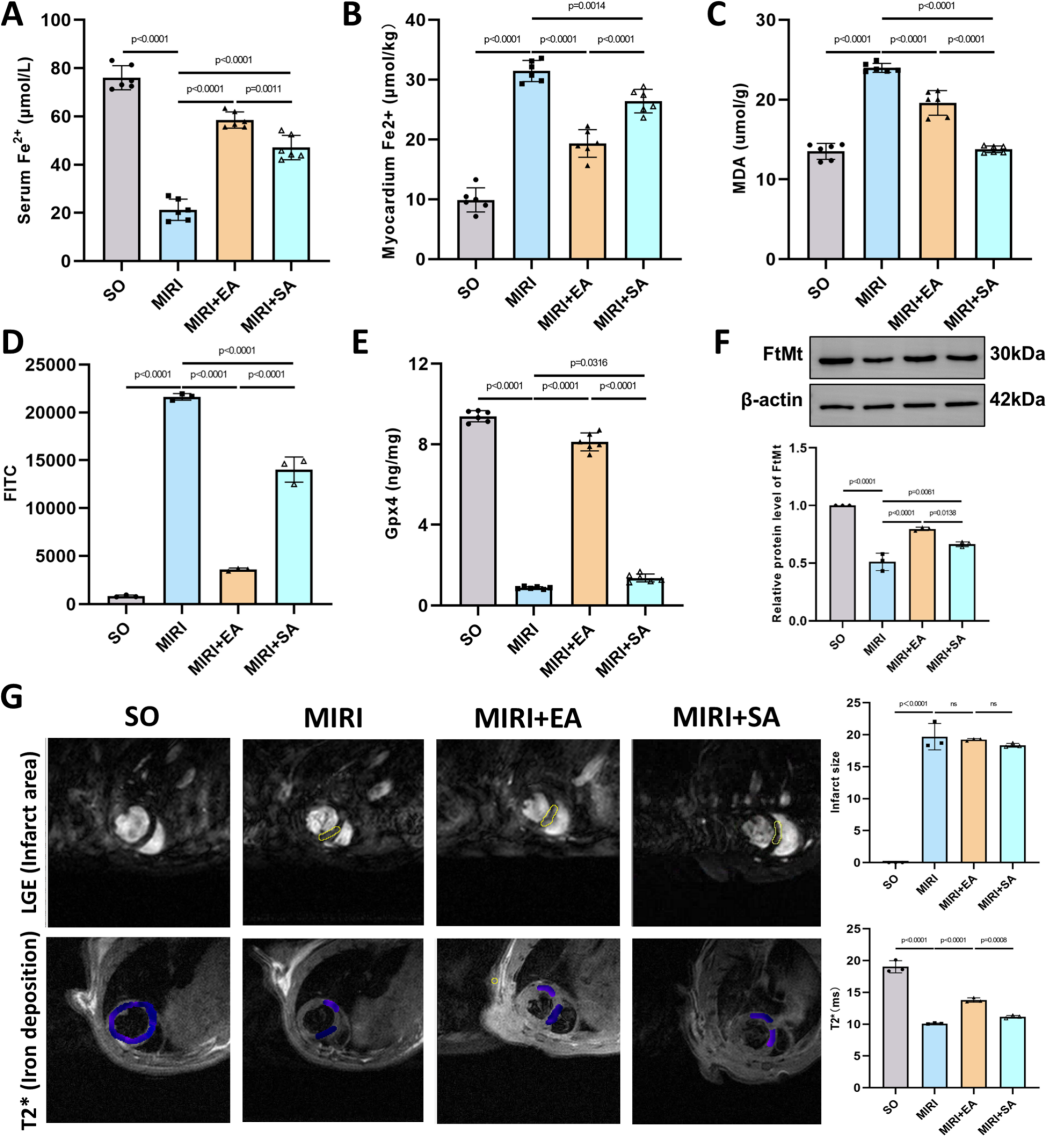
Acupuncture attenuates MIRI induced cardiomyocyte ferroptosis and reduce cardiac iron deposition. **A** Content of Fe^2+^ in serum of mice; n = 6; **B** Content of Fe^2+^ in myocardium of mice, n = 6; **C** Serum malondialdehyde (MDA) levels in mice; n = 6; **D** Expression of reactive oxygen species (ROS) in the myocardium of mice detected by flow cytometry; n = 6; **E** Content of glutathione peroxidase 4 (Gpx4) in myocardium of mice, n = 6; **F** Protein expression levels of mitochondrial ferritin (FtMt) in myocardial tissue, n = 3. **G** Representative magnetic resonance imaging (MRI) images of mice hearts in each group and quantitative results derived from the Late Gadolinium Enhancement (LGE) sequence and T2* sequence of MRI scans of mice’s hearts. The scanning results for all mice were processed and analyzed three times on the GE Advantage Workstation 4.6, with the average value taken as the final numerical result; n = 3. The yellow circles in the upper row images indicate areas of myocardial infarction and reperfusion. In the lower row, blue regions represent myocardial iron deposition, where higher T2* values signify less iron deposition. p values for all comparisons are indicated in the graph, with “ns” (non-significant) used for statistically insignificant results

Building on the observation that MIRI induces cardiomyocyte ferroptosis, we further investigated the therapeutic effects of acupuncture at PC6 on MIRI-induced ferroptosis. To explore the specific therapeutic effects of PC6, acupuncture at a sham point was used as control. The results showed that, compared with the MIRI group, serum Fe^2+^ levels were increased (Fig. 3A), and myocardial Fe^2+^ levels were decreased (Fig. 3B) in the MIRI + EA group. Additionally, levels of the lipid peroxidation product MDA (Fig. 3C) and intracellular ROS levels in cardiomyocytes (Fig. 3D) were significantly reduced in the MIRI + EA group. Furthermore, the therapeutic effects in the MIRI + EA group were superior to those in the MIRI + SA group (Fig. 3A–D). The ferroptosis markers Gpx4 and FtMt were decreased in MIRI group but significantly increased in the MIRI + EA group (Fig. 3E, F). Cardiac magnetic resonance imaging (MRI) was performed to visualise myocardial iron deposition. LGE sequence was used to assess the area of myocardial infarction, and no significant differences were observed between the MIRI, MIRI + EA, and MIRI + SA groups (Fig. 3G, upper panel). However, T2* sequence revealed that T2* values were lower in the MIRI group compared with the SO group, indicating increased myocardial iron deposition. T2* values were significantly higher in the MIRI + EA group than in the MIRI group and were also higher compared with the MIRI + SA group (Fig. 3G, lower panel). These findings suggest that myocardial iron deposition increased in the MIRI group compared with the SO group, while EA at PC6 reduced iron deposition more effectively than SA.

### Acupuncture alleviates MIRI and protects cardiac function

**Fig. 4:**
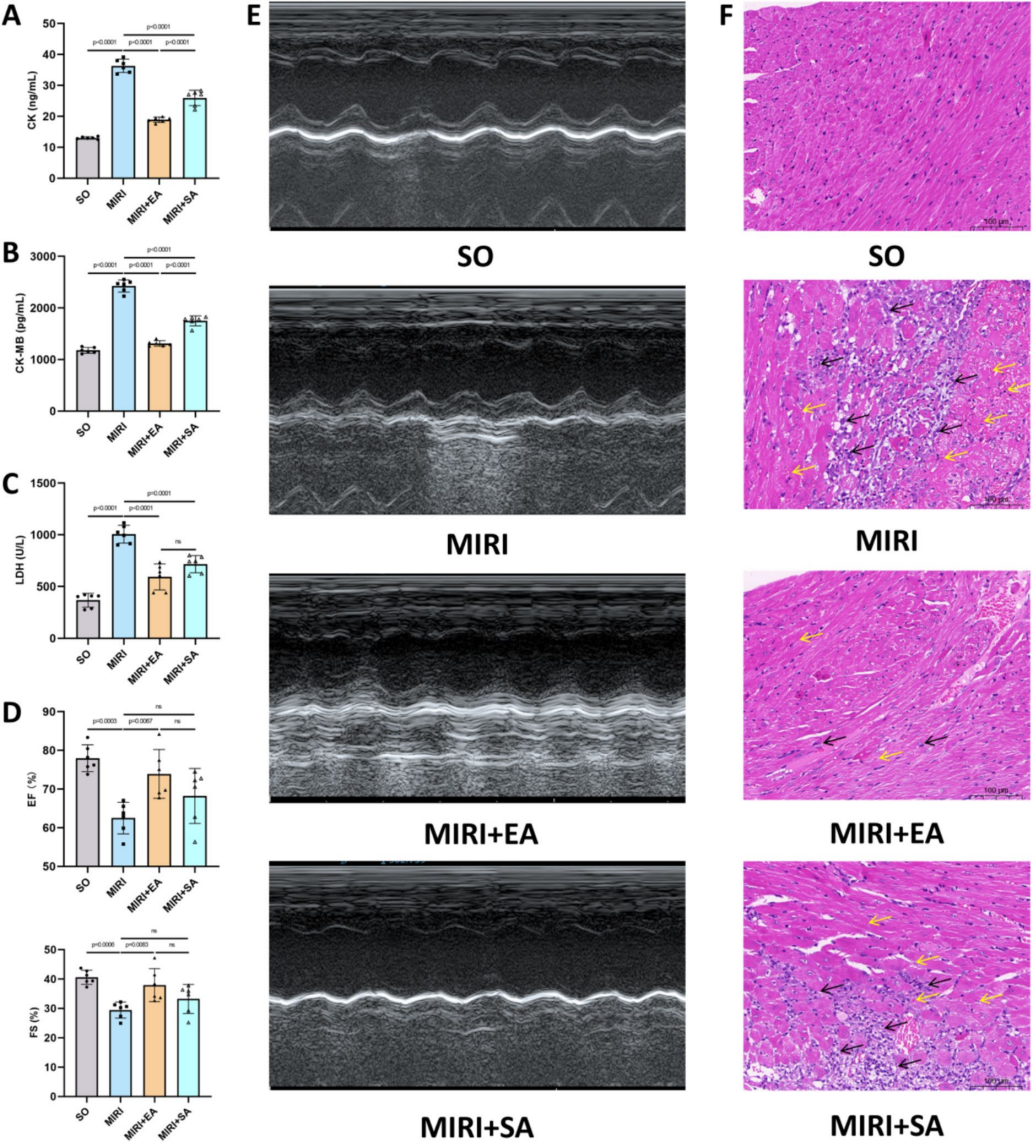
Acupuncture exerts protective effects on MIRI. **A**–**C** Content of CK, CK-MB and LDH in serum of mice; n = 6; **D** Left ventricular ejection fraction (EF) and fractional shortening (FS) of mice by cardiac ultrasound. The final result for each mouse was determined by averaging EF and FS measurements across three cardiac cycles; n = 6; **E** Representative cardiac ultrasound images of mice from each group; **F** Representative myocardium Hematoxylin–eosin (HE) staining images of mice from each group (100 ×, scale bar = 100 μm). p values for all comparisons are indicated in the graph, with “ns” (non-significant) used for statistically insignificant results

The ultimate goal of treating MIRI is to improve cardiac function. Therefore, we further evaluated the therapeutic effects of acupuncture on MIRI and its protective role in cardiac function. Compared with the MIRI group, serum levels of the myocardial enzymes CK, CK-MB, and LDH were significantly reduced in the MIRI + EA group (Fig. 4A–C). CK and CK-MB levels in the MIRI + EA group showed greater improvement compared with the MIRI + SA group (Fig. 4A, B), although no significant difference in LDH levels was observed between the two groups (Fig. 4C). Echocardiographic analysis revealed that, compared with the MIRI group, both EF and FS values were significantly increased in the MIRI + EA group. While the MIRI + EA group showed a trend towards better outcomes than the MIRI + SA group, the differences were not statistically significant (Fig. 4D). HE staining was used to observe the morphology of cardiomyocytes. In the MIRI group, disordered myocardial fibre arrangement, significant inflammatory cell infiltration (black arrows), and necrosis of individual cardiomyocytes (yellow arrows) were observed. These pathological changes were noticeably improved following EA treatment, with EA showing superior effects compared to SA (Fig. 4F). Given that increased ROS and ferroptosis following MIRI can lead to elevated levels of inflammatory cytokines, we measured the myocardial levels of IL-1β, IL-6, and TNF-α. The trends observed in these inflammatory cytokines were consistent with the changes in myocardial enzyme levels (Supplementary Fig. 1).

### Acupuncture may regulate ferroptosis via the Nrf2/HO-1 pathway

**Fig. 5:**
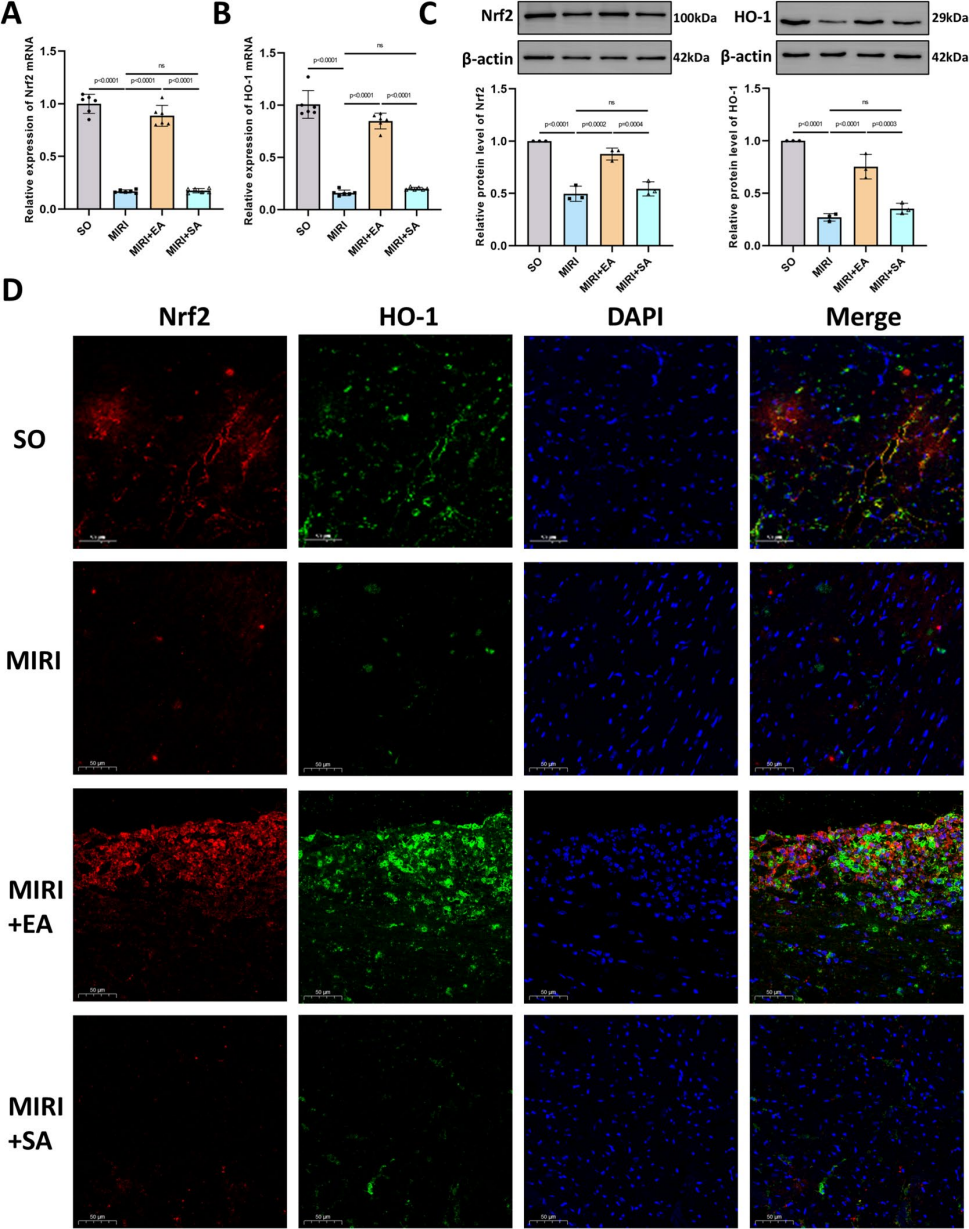
Acupuncture exerts a protective effect on MIRI by regulating ferroptosis via the Nrf2/HO-1 pathway. **A** Nrf2 mRNA levels in myocardial tissue of mice in each group; n = 6; **B** HO-1 mRNA levels in myocardial tissue of mice in each group; n = 6; **C** Protein expression levels of Nrf2 and HO-1 in myocardial tissue, n = 3; **D** Representative images of double immunofluorescence staining for Nrf2 (red) and HO-1 (green). Cell nuclei were stained with DAPI (blue). 400 ×, scale bar = 50 μm. p values for all comparisons are indicated in the graph, with “ns” (non-significant) used for statistically insignificant results

Previous studies have demonstrated that acupuncture can upregulate the Nrf2/HO-1 pathway to alleviate oxidative stress or ferroptosis [14, 26, 27]. Therefore, we hypothesised that acupuncture may regulate ferroptosis via the Nrf2/HO-1 pathway to mitigate MIRI and improve cardiac function. We examined the protein and mRNA expression levels of Nrf2 and HO-1 in cardiac tissue. RT-qPCR results revealed that mRNA levels of Nrf2 and HO-1 were significantly reduced in the MIRI group compared with the SO group, while these levels were significantly increased in the MIRI + EA group compared with the MIRI group. Furthermore, the MIRI + EA group exhibited superior effects compared with the MIRI + SA group (Fig. 5A, B). Western blotting showed a similar trend in the protein expression of Nrf2 and HO-1 in myocardial tissue (Fig. 5C). Double immunofluorescence staining of Nrf2 and HO-1 was performed, and quantitative analysis of the positive expression levels showed the same trend (Fig. 5D, Supplementary Fig. 2). Overall, these results suggest that EA intervention increased the expression of Nrf2 and HO-1, which were otherwise reduced by MIRI.

### The Nrf2 inhibitor partially blocks the anti-ferroptosis and cardioprotective effects of acupuncture, suggesting that acupuncture exerts its protective effects on MIRI via the Nrf2/HO-1 pathway

**Fig. 6:**
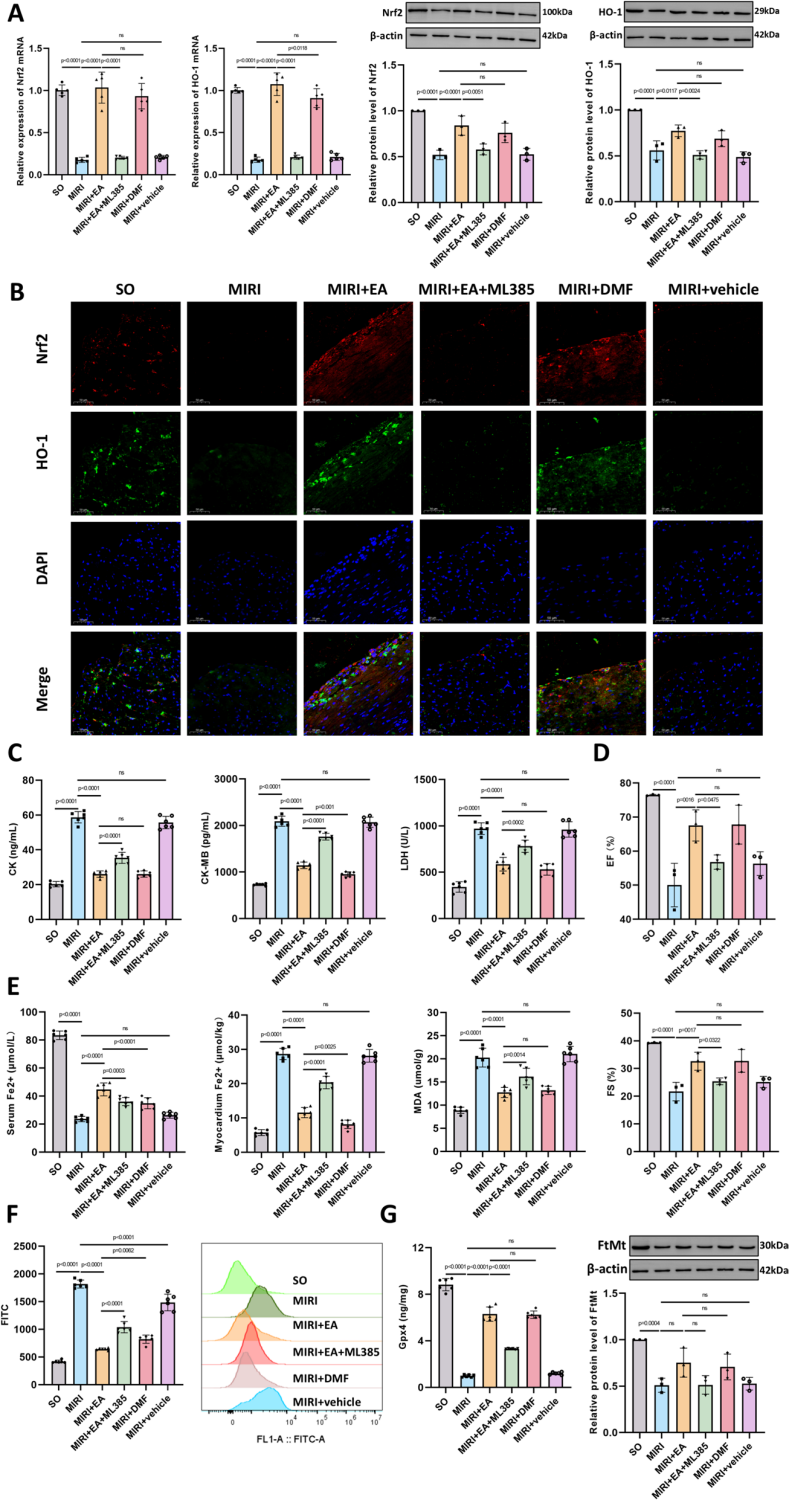
ML385 partially blocks the therapeutic effect of acupuncture on ferroptosis and cardiac function, suggesting that acupuncture exerts a protective effect on MIRI by inhibiting ferroptosis via the Nrf2/HO-1 pathway. **A** mRNA (n = 6) and protein (n = 3) levels of Nrf2 and HO-1 in myocardial tissue of mice in each group; **B** Representative images of double immunofluorescence staining of Nrf2 (red) and HO-1 (green). Cell nuclei were counterstained with DAPI (blue). 400 ×; scale bar = 50 µm; **C** Serum levels of CK, CK-MB, and LDH in mice in each group, n = 6; **D** Echocardiographic measurements of left ventricular ejection fraction (EF) and fractional shortening (FS) in mice in each group, n = 3; **E** Serum Fe^2^⁺, myocardial Fe^2^⁺, serum malondialdehyde (MDA) levels in mice in each group, n = 6; **F** Myocardial reactive oxygen species (ROS) levels in mice in each group, n = 6; **G** Glutathione peroxidase 4 (Gpx4) levels (n = 6) and protein levels of mitochondrial ferritin (FtMt) (n = 3) in myocardial tissue. p values for all comparisons are indicated in the graph, with “ns” (non-significant) used for statistically insignificant results

To further verify that acupuncture primarily alleviates MIRI and improves cardiac function through the Nrf2/HO-1 pathway, we utilised an Nrf2 inhibitor (ML385) and an Nrf2 activator (DMF). Following Nrf2 inhibition with ML385, the expression of Nrf2 and HO-1 proteins and mRNA was significantly reduced in the MIRI + EA + ML385 group compared with the MIRI + EA group. The expression of Nrf2 and HO-1 proteins and mRNA in the MIRI + EA group showed a similar trend to that in the MIRI + DMF group, indicating that EA treatment had a similar effect to DMF in upregulating and activating Nrf2 and its downstream target HO-1 (Fig. 6A). Double immunofluorescence staining of Nrf2 and HO-1 yielded results consistent with the qPCR and WB findings (Fig. 6B).

The results showed that compared with the MIRI + EA group, myocardial enzyme levels were significantly elevated in the MIRI + EA + ML385 group (Fig. 6C), and serum inflammatory cytokines were markedly increased (Supplementary Fig. 3). Additionally, EF and FS values measured by cardiac ultrasound were significantly decreased in the MIRI + EA + ML385 group compared with the MIRI + EA group (Fig. 6D). The MIRI + DMF group exhibited a similar trend to the MIRI + EA group in terms of myocardial enzyme levels, serum inflammatory cytokines, and EF/FS values (Fig. 6C, D, Supplementary Fig. 3). These findings suggest that EA treatment exerted a DMF-like effect, and the cardioprotective effects of EA could be blocked by the Nrf2 inhibitor.

Compared with the MIRI + EA group, the MIRI + EA + ML385 group exhibited reduced serum Fe^2+^ levels, increased myocardial Fe^2+^ levels, significantly elevated serum MDA levels, increased myocardial ROS levels as well as decreased Gpx4 and FtMt levels (Fig. 6E–G). The MIRI + DMF group showed similar trends to the MIRI + EA group in serum Fe^2+^, myocardial Fe^2+^, serum MDA, and myocardial ROS, Gpx4 and FtMt levels (Fig. 6E–G). These results indicate that EA treatment exerted an anti-ferroptotic effect similar to that of DMF, and the improvement in iron deposition by EA could be blocked by the Nrf2 inhibitor.

## Discussion

Ferroptosis of cardiomyocytes represents a significant pathological process contributing to the damage induced by MIRI [2, 3]. The findings of this study once again demonstrate that MIRI can significantly induce ferroptosis in cardiomyocytes. Moreover, selecting the specific acupoint PC6 not only significantly reduces the degree of ferroptosis in cardiomyocytes and effectively decreases iron deposition in cardiac tissue, but also markedly alleviates the adverse effects of MIRI on heart function, exerting a protective effect on the heart. By utilizing Nrf2 inhibitors and agonists, this study initially confirms that acupuncture primarily exerts its effects through the Nrf2/HO-1 pathway. Compared to a previous work [28], the current study further advances the understanding of acupuncture's therapeutic mechanisms by incorporating a more rigorous experimental design, advanced imaging techniques such as cardiac MRI, and comprehensive mechanistic validation using both Nrf2 inhibitors and activators. These innovations provide a deeper insight into the role of acupuncture in mitigating MIRI-induced ferroptosis and highlight its potential as a preventive intervention.

Unlike chemical drugs, which have strict time restrictions for intervention, acupuncture has a more flexible timing for intervention. This flexibility allows for acupuncture treatment to be administered at any stage of the disease process, thereby providing an opportunity to enhance the therapeutic effects as an adjunctive treatment. Taking into account the irreversibility of cardiomyocyte death, this study employed an acupuncture pretreatment strategy, which involves treatment administered prior to the onset of disease. Actually, the acupuncture pretreatment strategy is widely employed in the mechanistic study of MIRI [29–31]. This strategy confers significant advantages in mechanistic research, as it allows for comprehensive monitoring of relevant indices throughout the entire process of MIRI. From a mechanistic standpoint, acupuncture pretreatment provides a crucial theoretical foundation for subsequent clinical interventions. Elucidating the mechanisms underlying acupuncture pretreatment can pave the way for the development of more effective therapeutic strategies, thereby offering greater potential for clinical applications in the treatment of MIRI. However, it is evident that the pretreatment regimen cannot be applied to all MIRI patients in clinical settings, as procedures are categorized into elective percutaneous coronary intervention (PCI) surgeries and urgent surgeries that must be performed immediately. For the latter group, acupuncture pretreatment is impractical due to the time constraints, thus the conclusions of this study cannot be directly applied to such patients, which restricts the clinical translational value of the research findings. Future research endeavors will be directed toward a comprehensive elucidation of the therapeutic efficacy and underlying mechanisms of acupuncture in the context of MIRI. Specifically, this will involve investigating the effects of acupuncture administered both after MIRI modeling and during the interval between myocardial ischemia and reperfusion, in order to identify more appropriate and efficient timing for acupuncture intervention when it’s possible to choose. Furthermore, given that the current study has primarily focused on short-term effects, the incorporation of long-term follow-up assessments will enable the evaluation of sustained therapeutic effects, with a focus on critical observational indices, including cardiac remodeling, cardiomyocyte survival rate, and alterations in cardiac function, and the incidence of arrhythmias following reperfusion. These multifaceted approaches are anticipated to address the limitations inherent in our current study and contribute to a more holistic and scientifically robust understanding of acupuncture's therapeutic potential across both the acute and chronic phases of MIRI.

Ferroptosis is a novel form of programmed cell death characterized by iron-dependent lipid peroxidation [32]. Consistent with previous research findings, during MIRI, cardiomyocytes undergo severe oxidative stress damage, leading to elevated intracellular iron levels, which subsequently triggers lipid peroxidation and ultimately results in cardiomyocyte death [33, 34]. This study discovered that acupuncture therapy can significantly reduce both intracellular iron levels and the degree of lipid peroxidation in cardiomyocytes, thereby mitigating ferroptosis-induced damage to these cells. These results align with previous findings regarding the anti-ferroptotic mechanisms of acupuncture. Furthermore, in this study, to clearly demonstrate acupuncture's regulatory effect on iron deposition in myocardial tissue, we employed cardiac MRI techniques. The LGE sequence was used to clearly visualize the area of myocardial infarction, while the T2* sequence enabled quantification of iron content in the myocardium, thus providing a more accurate assessment of the improvement in myocardial iron deposition achieved through acupuncture therapy [35]. Cardiac MRI is non-invasive and radiation-free, allowing for repeated and continuous monitoring of cardiac changes without affecting the physiological state of mice. This enables a more precise evaluation of the long-term interventional effects of acupuncture therapy on the ferroptosis process in cardiomyocytes and provides crucial evidence for future clinical decision-making. This represents a bold attempt to apply interdisciplinary experimental techniques to validate acupuncture treatment targets. However, cardiac MRI currently has disadvantages such as high cost and long imaging times. In the future, it is anticipated that innovative technologies, optimized scanning sequences, and enhanced interdisciplinary collaboration will jointly drive the development and application of cardiac MRI.

The Nrf2/HO-1 pathway is a crucial antioxidant stress pathway that plays a pivotal role in regulating cellular antioxidant capacity. Nrf2, a transcription factor, translocates from the cytoplasm to the nucleus when cells are subjected to oxidative stress damage. In the nucleus, it binds to ARE and initiates the synthesis of a series of antioxidant enzymes, such as HO-1 [16, 36, 37]. HO-1, a heme oxygenase, catalyzes the decomposition of heme into biliverdin, carbon monoxide, and iron ions, thereby mitigating intracellular oxidative stress damage [17]. Activation of Nrf2 can inhibit ferroptosis by enhancing cellular antioxidant capacity [38], reducing ROS levels, and upregulating Gpx4 mRNA expression [39, 40]. Furthermore, activation of the Nrf2/HO-1 signaling pathway reduces ferroptosis in various cell types, including cardiomyocytes [41–43]. Previous studies have shown that acupuncture exerts anti-inflammatory and antioxidant effects through the Nrf2/HO-1 signaling pathway in diseases such as inflammatory bowel disease [44], ulcerative colitis [45], diabetic encephalopathy [46], and lung injury [47], and vascular dementia [48]. In this study, we investigated the effects of acupuncture on Nrf2 and HO-1 in MIRI and found that acupuncture treatment significantly upregulated the expression levels of both Nrf2 and HO-1. This suggests that acupuncture may enhance the antioxidant capacity of cardiomyocytes and mitigate ferroptosis damage by activating the Nrf2/HO-1 pathway. Further research has revealed that Nrf2 inhibitors, such as ML385 can partially block the anti-ferroptotic and cardioprotective therapeutic effects of acupuncture. ML385 specifically targets the Nrf2 protein, preventing its translocation to the nucleus and subsequent binding to the ARE, thereby inhibiting the transcription of downstream antioxidant genes like HO-1 [49]. This mechanism underscores the critical role of the Nrf2/HO-1 pathway in acupuncture treatment for MIRI-induced ferroptosis damage. However, while the use of Nrf2 inhibitors and agonists can provide certain evidence, there are still limitations in terms of the accuracy of pathway validation. Future studies may consider using Nrf2 or HO-1 gene knockout mouse models to further verify the mechanism of action of acupuncture. Furthermore, the Nrf2/HO-1 pathway may only be part of a complex signaling network. In real-world human settings, influenced by various factors such as medication and disease duration, changes in related pathways and potential interactions may require further exploration. Future research should aim to comprehensively understand the molecular mechanisms underlying acupuncture's protection of cardiac function by delving into the upstream and downstream molecular mechanisms of Nrf2/HO-1 pathway or by integrating more complex pathways related to antioxidant and anti-ferroptotic activities. This will also provide insights for the development of new therapeutic strategies.

Nevertheless, this research on the inhibitory effect of acupuncture on ferroptosis did not include a ferroptosis inhibitor as a positive control. The absence of such a control group may have limited the interpretability of the results, as direct comparisons with established ferroptosis inhibitors (e.g., ferrostatin-1 or liproxstatin-1) could have provided more robust evidence to distinguish the specific anti-ferroptotic effects of acupuncture from general cytoprotective mechanisms. Future studies should incorporate pharmacological inhibitors of ferroptosis to validate the targeted molecular pathways and strengthen the conclusions regarding acupuncture's therapeutic role in mitigating iron-dependent cell death. Additionally, this study employed a relatively small number of MIRI mice to investigate the effects of acupuncture on ferroptosis and cardiac function following MIRI. Future research is recommended to increase the sample size to enhance statistical power and to utilize a broader range of animal models and varied intervention times. This approach would help verify the effects of acupuncture at different stages of the disease and across different disease contexts.

## Conclusion

In summary, this study reveals that acupuncture pre-treatment alleviates MIRI-induced ferroptosis by activating the Nrf2/HO-1 pathway, providing mechanistic evidence for its cardioprotective effects. These findings offer modern scientific evidence for the application of acupuncture in the treatment of cardiovascular diseases. Future research will aim to deepen and refine these findings, contributing to the broader application of acupuncture in the treatment of cardiovascular diseases.

## Supplementary Information

Additional file 1

## Data Availability

The data used to support the findings of this study are available from the corresponding author upon request.

## Abbreviations

ARE: Antioxidant response element
CK: Creatine kinase
CK-MB: Creatine kinase-MB
ddH₂O: Double-distilled water
DMF: Dimethyl fumarate
DMSO: Dimethyl sulfoxide
EA: Electro-acupuncture
EF: Ejection fraction
ELISA: Enzyme-linked immunosorbent assay
FS: Fraction shorting
FtMt: Mitochondrial ferritin
Gpx4: Glutathione peroxidase 4
HE: Hematoxylin–eosin
HO-1: Heme oxygenase-1
IL-1β: Interleukin-1β
IL-6: Interleukin-6
IR: Ischemia/reperfusion
LCA: Left coronary artery
LDH: Lactate dehydrogenase
LGE: Late gadolinium enhancement
MDA: Malondialdehyde
MIRI: Myocardial ischemia/reperfusion injury
MRI: Magnetic resonance imaging
Nrf2: Nuclear factor-E2-related factor
PCR: Polymerase chain reaction
PEG300: Polyethylene glycol 300
ROS: Reactive oxygen species
SA: Sham acupuncture
SD: Standard deviation
SO: Sham operation
SPF: Specific pathogen-free
TNF-α: Tumour necrosis factor-α
