# Supplementary material for: Acupuncture attenuates myocardial ischemia/reperfusion injury-induced ferroptosis via the Nrf2/HO-1 pathway
Source: Chin Med. 2025 May 9;20:61. doi: 10.1186/s13020-025-01114-0 (PMC12065278; doi:10.1186/s13020-025-01114-0)
Supplement: Supplementary file 1 — Additional file 1 [file 13020_2025_1114_MOESM1_ESM.docx]

**Supplementary material**


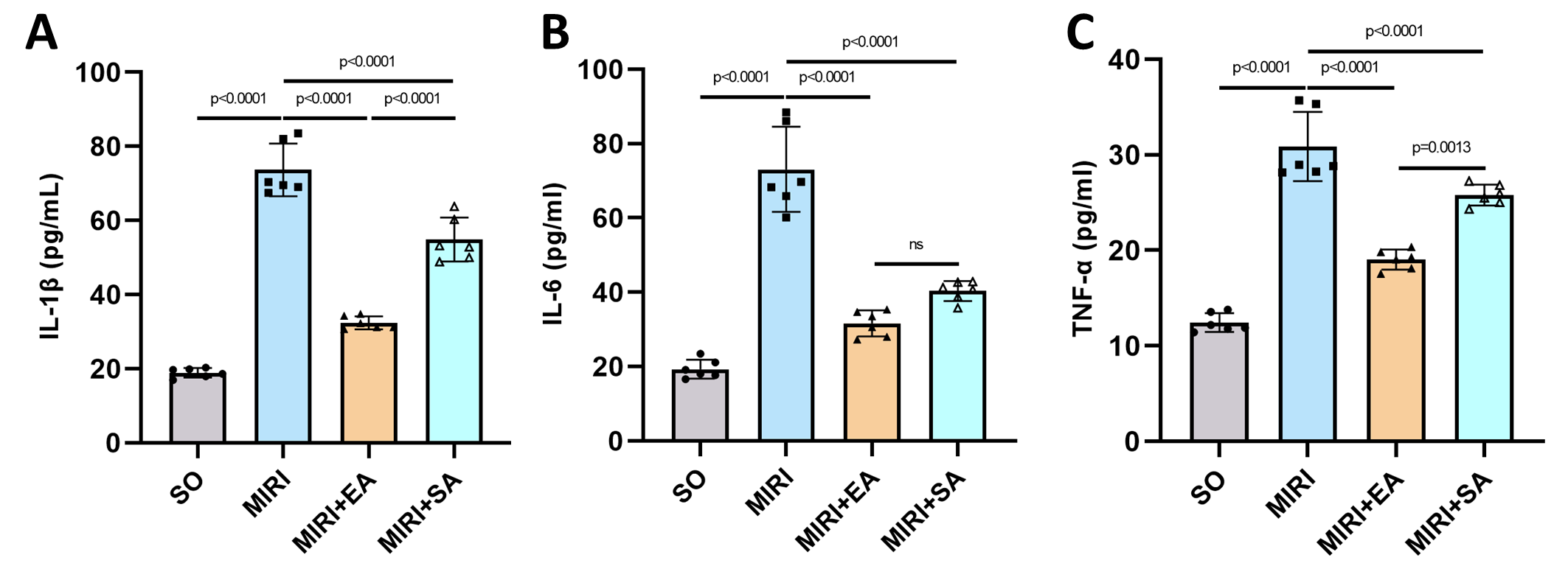


Supplementary Figure 1. Serum inflammatory factor expression. (A) Content of IL-1β in serum of mice; n=6 per group; (B) Content of IL-6 in serum of mice; n=6 per group; (C) Content of TNF-α in serum of mice; n=6 per group. p values for all comparisons are indicated in the graph.


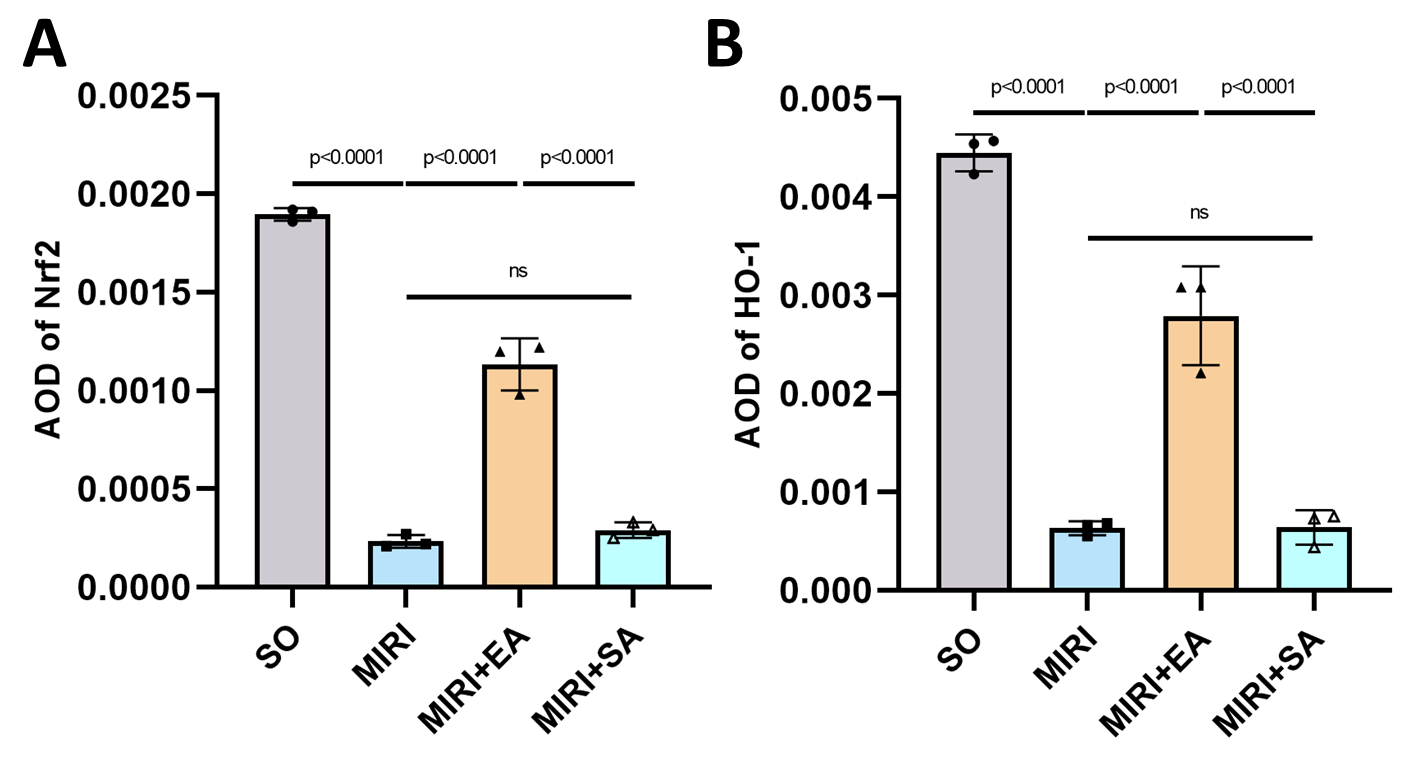


Supplementary Figure 2. Quantitative analysis of the positive expression levels for Nrf2 (A) and HO-1 (B). p values for all comparisons are indicated in the graph.


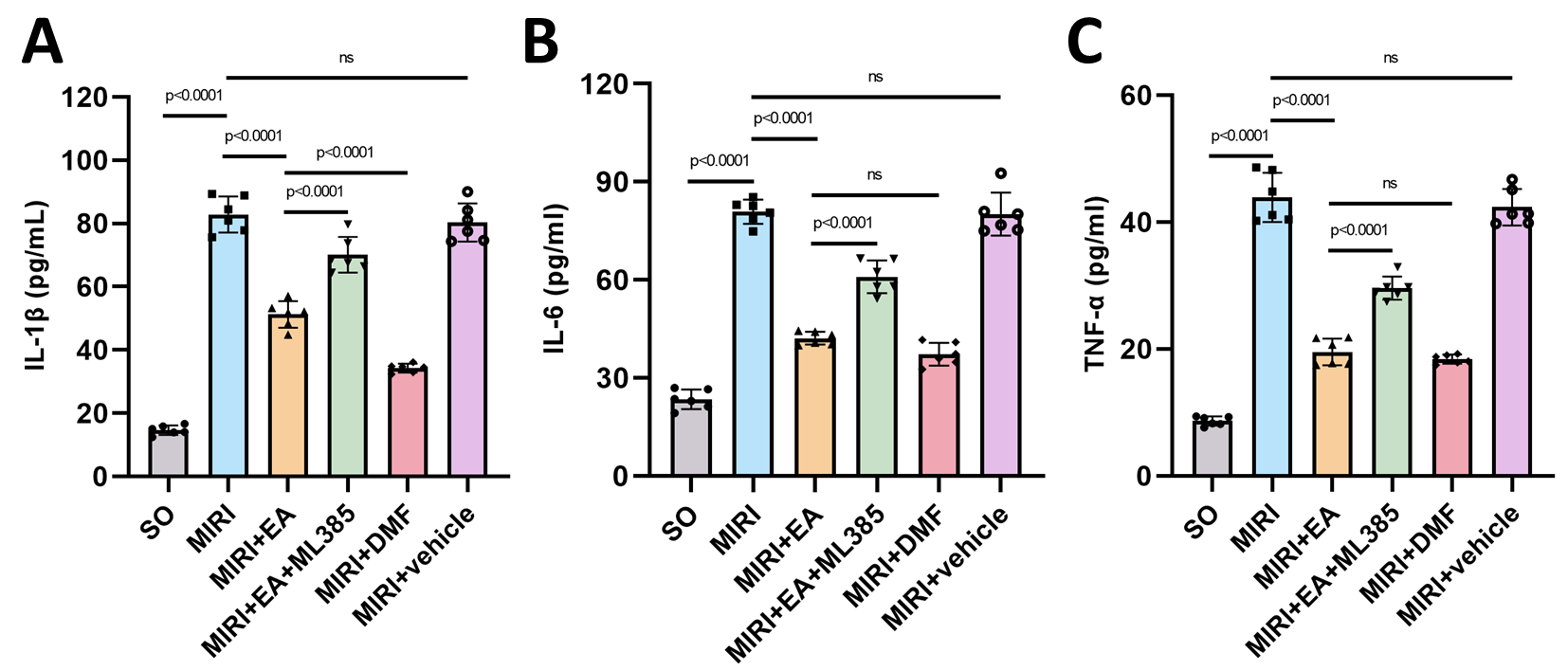


Supplementary Figure 3. Serum inflammatory factor expression. (A) Content of IL-1β in serum of mice; n=6 per group; (B) Content of IL-6 in serum of mice; n=6 per group; (C) Content of TNF-α in serum of mice; n=6 per group. p values for all comparisons are indicated in the graph.
